# Early midazolam exposure and short-term risk of acute exacerbation of COPD in critically ill patients: a target trial emulation using the MIMIC-IV database

**DOI:** 10.3389/frai.2026.1836581

**Published:** 2026-10-06

**Authors:** Guoping Qin, Kun Zou

**Affiliations:** Department of Respiratory and Critical Care Medicine, Dongguan Qingxi Hospital, Dongguan, China

**Keywords:** acute exacerbation, clone-censor-weight, COPD, inverse probability of censoring weighting, midazolam, MIMIC-IV, target trial emulation

## Abstract

**Background:**

Sedation is frequently required in the intensive care unit (ICU), but benzodiazepines may worsen respiratory outcomes in patients with chronic obstructive pulmonary disease (COPD).

**Objective:**

To evaluate whether early midazolam exposure after ICU admission is associated with the short-term risk of acute exacerbation of COPD (AECOPD) among critically ill COPD patients.

**Methods:**

Using the Medical Information Mart for Intensive Care IV (MIMIC-IV) database, we emulated a target trial and applied a clone-censor-weight (CCW) approach to address immortal time bias and informative censoring. Exposure was midazolam administration within a grace period after ICU admission (primary: 24 h; sensitivity: 12 and 36 h). The primary outcome was AECOPD, defined as initiation of systemic corticosteroids or antibiotics between 24 h and 7 days after ICU admission. Outcomes were compared using inverse probability of censoring weights (IPCW).

**Results:**

Among 3,416 ICU stays, 233 (6.8%) received midazolam within 24 h. After IPCW weighting, AECOPD incidence was 29.8% in the midazolam group versus 23.5% in controls (risk difference +6.3%, 95% CI −2.7% to +15.8%; odds ratio 1.38, 95% CI 0.86–2.12; *p* = 0.158). Findings were directionally consistent across grace periods (12 h OR 1.64; 36 h OR 1.29), but none of the grace-period comparisons reached statistical significance.

**Conclusion:**

In the primary 24 h analysis, early midazolam exposure was associated with a numerically higher short-term AECOPD risk. These observational findings cannot establish causality, and the present study did not demonstrate a statistically significant causal effect; nevertheless, in view of existing ICU sedation evidence, continued caution in the use of midazolam in critically ill COPD patients appears prudent.

## Introduction

Chronic obstructive pulmonary disease (COPD) is a leading cause of morbidity and mortality worldwide and remains a major public health burden (Agusti and Vogelmeier, 2023; Cao et al., 2026; Vogelmeier et al., 2017). Acute exacerbations are a key driver of health-care utilization, often prompting hospitalization and repeated readmissions, and are associated with substantial short- and long-term mortality risk (Landt et al., 2024; Ruan et al., 2023; Waeijen-Smit et al., 2024). A proportion of patients deteriorate to acute hypercapnic respiratory failure and require escalation to intensive care for ventilatory support; evidence-based respiratory support strategies (including non-invasive ventilation in appropriate patients) can improve outcomes, but many patients still require invasive mechanical ventilation (Vogelmeier et al., 2017; Nevins and Epstein, 2001; Plant et al., 2000). In the ICU, COPD is frequently complicated by severe dyspnea, agitation, altered mental status, and patient–ventilator desynchrony, and may coexist with infectious triggers such as community-acquired pneumonia, further increasing physiologic stress and complexity of care (Vogelmeier et al., 2017; Rello et al., 2006). Consequently, many critically ill COPD patients receive continuous sedation to facilitate safe ventilation and delivery of ICU therapies (Devlin et al., 2018). However, sedation in COPD is clinically challenging because sedatives can depress respiratory drive, worsen hypercapnia, impair airway protective reflexes, and prolong ventilator dependence, which may be particularly consequential in patients predisposed to chronic hypercapnia.

Benzodiazepines, including midazolam, remain widely used in ICUs because of their rapid onset, titratability, and familiarity (Devlin et al., 2018). At the same time, contemporary ICU sedation guidelines emphasize minimizing benzodiazepine exposure when feasible and recommend alternative agents in many settings (Devlin et al., 2018). These recommendations are supported by evidence linking benzodiazepine exposure to delirium and by systematic reviews suggesting less favorable ICU course compared with non-benzodiazepine strategies (Fraser et al., 2013; Pandharipande et al., 2006). For patients with COPD, concerns about benzodiazepines may be amplified because even small decrements in ventilatory drive and upper airway tone can precipitate carbon dioxide retention and dynamic hyperinflation, thereby increasing the risk of clinical deterioration (Vogelmeier et al., 2017). Despite biologic plausibility and guideline concerns, evidence directly addressing whether midazolam administration in the ICU may aggravate COPD patients—particularly by increasing the risk of acute exacerbation events shortly after ICU admission—remains limited (Devlin et al., 2018).

A major challenge in evaluating the effects of early ICU medications using observational data is the presence of time-related biases and confounding by indication. Midazolam is often initiated after ICU admission in response to evolving severity, procedures, or ventilator needs; consequently, naïve comparisons of “ever” versus “never” exposure can introduce immortal time bias and selection bias (Hernán and Robins, 2016). Target trial emulation provides a principled framework to define eligibility, treatment strategies, time zero, follow-up, and outcomes in a way that mimics a randomized trial, thereby clarifying the causal question and reducing avoidable biases (Hernán and Robins, 2016). In the context of treatment initiation within a grace period after ICU admission, clone–censor–weight (CCW) methods can further approximate per-protocol contrasts by cloning patients, censoring strategy-inconsistent clones, and weighting to account for informative censoring (Hernán and Robins, 2016; Gaber et al., 2024).

The present study therefore aimed to evaluate whether midazolam administration in the ICU is associated with short-term worsening among critically ill COPD patients. Specifically, we examined whether initiating midazolam within a pre-specified grace period after ICU admission was associated with the risk of acute exacerbation of COPD (AECOPD) during the subsequent 7 days. We emulated a target trial using the Medical Information Mart for Intensive Care IV (MIMIC-IV) database (Reep et al., 2025), using a CCW approach to mitigate immortal time bias and informative censoring (Hernán and Robins, 2016; Gaber et al., 2024).

## Methods

### Study design and data source

This study was designed as a target trial emulation (TTE) using observational ICU data (Hernán and Robins, 2016; Hernán et al., 2022; Matthews et al., 2022). Reporting follows the STROBE guidelines (von Elm et al., 2007) and the TARGET guideline for transparent reporting of observational studies emulating a target trial (Cashin et al., 2025). Data were obtained from the Medical Information Mart for Intensive Care IV (MIMIC-IV) database (version 3.1), a large de-identified critical care dataset from Beth Israel Deaconess Medical Center covering 2008 to 2019 (Johnson et al., 2023; Xu et al., 2023). The database provides longitudinal ICU information including demographics, diagnoses, physiological measurements, laboratory results, medication prescriptions and administrations, procedures, and outcomes.

### Ethics statement

This study is a secondary analysis of de-identified data from the MIMIC-IV database (version 3.1). The original data collection was approved by the Institutional Review Boards of the Massachusetts Institute of Technology and Beth Israel Deaconess Medical Center (IRB protocol #2001P001699), with a waiver of informed consent granted. All methods were performed in accordance with relevant guidelines and regulations. Analysis code is available upon reasonable request.

### Study population

The source population consisted of adult patients (≥18 years) with a recorded COPD diagnosis during the index hospitalization, identified using ICD-10 code J44.x (Vogelmeier et al., 2017; Thomsen et al., 2011). We included all ICU stays meeting the eligibility criteria; a sensitivity analysis restricted to the first ICU stay per patient is presented to address within-patient correlation (the primary analysis accounts for clustering through patient-level bootstrap resampling). We required an ICU length of stay greater than 24 h to ensure adequate time for exposure assessment and outcome ascertainment within the pre-specified windows. We excluded patients missing critical baseline variables because these covariates were required for weighting and risk adjustment. To ensure a clear temporal sequence between ICU admission and exposure, patients who received midazolam prior to ICU admission were excluded; this also operationalizes a new-user design to reduce bias related to prevalent (current) users (Hernán, 2018; Li et al., 2025). To reduce outcome misclassification and to avoid including patients with a likely pre-existing exacerbation at admission, we excluded patients who initiated systemic corticosteroids or antibiotics within the first 24 h after ICU admission. After applying these criteria, 3,416 ICU stays (from 2,750 patients) comprised the final analytical cohort.

### Exposure definition

Midazolam exposure was defined as administration of midazolam by intravenous infusion or injection within a pre-specified grace period after ICU admission. Exposure was identified from the *inputevents* administration records (item IDs 221668 and 225150); a sensitivity analysis additionally incorporated prescription records (union definition). Patients were classified as exposed if they received midazolam within the grace period and as unexposed otherwise.

The 24 h grace period was chosen as the primary window because it corresponds to the conventional first ICU day, during which sedation is typically initiated in response to early clinical deterioration, and it aligns with the start of the outcome-ascertainment window (24 h to 7 days). The 12 h and 36 h windows bracket the primary window to evaluate sensitivity to exposure timing: the 12 h window captures very early sedation initiation (e.g., at intubation), whereas the 36 h window accommodates delayed initiation after a period of observation.

### Outcome definition

The primary outcome was AECOPD, defined as initiation of systemic corticosteroids or antibiotics between 24 h and 7 days after ICU admission. Systemic corticosteroids included prednisone, methylprednisolone, dexamethasone, or hydrocortisone. Antibiotics included amoxicillin, levofloxacin, azithromycin, ceftriaxone, cefuroxime, moxifloxacin, or doxycycline. The 24 h post-admission exclusion window was used to reduce misclassification of medications initiated for indications other than AECOPD. Secondary outcomes included 7-day mortality, 28-day mortality, 7-day survival, ICU length of stay (LOS), survival time from ICU admission to death or ICU discharge (censored at 7 days), restricted mean survival time (RMST) at 7 days, and restricted mean time lost (RMTL) at 7 days (Cai et al., 2022; Diop et al., 2021). Mortality was ascertained from the recorded time of death.

### Statistical analysis

We conducted the analysis under a target trial emulation framework (Hernán and Robins, 2016; Hernán et al., 2022; Matthews et al., 2022). Because treatment intention is not observed in routinely collected ICU data, the primary causal contrast corresponds to a per-protocol effect; CCW methods were used to mitigate immortal time bias in the setting of delayed treatment initiation (Gaber et al., 2024; Li et al., 2025; Yuan et al., 2020). The CCW approach was implemented in three steps. First, each ICU stay was cloned into two copies, one assigned to a strategy of initiating midazolam within the grace period and the other assigned to a strategy of not initiating midazolam within the grace period. Second, clones were censored when their observed treatment deviated from the assigned strategy: control-strategy clones were censored at the time midazolam was actually initiated within the grace period, whereas treatment-strategy clones were censored at the end of the grace period if midazolam was not initiated. Third, we applied inverse probability of censoring weights (IPCW) to account for informative censoring (Gaber et al., 2024; Li et al., 2025). Because the censoring mechanism differs fundamentally between strategies, censoring models were fitted separately within each strategy (per-arm models) using logistic regression. For each uncensored clone, the stabilized weight was defined as

$$w_{i}=\frac{P(C_{i}=0|A_{i})}{P(C_{i}=0|X_{i},A_{i})}$$

Where *C* denotes the censoring indicator, *A* the assigned strategy, and *X* the baseline covariates; censored clones received zero weight. Weights were truncated at the 99th percentile of the uncensored weights within each strategy to reduce the impact of extreme values. The TTE protocol was summarized in Supplementary Table 2 and the positivity assessment, the love plot and the IPCW weight distribution were shown in Supplementary Figures 1–3.

Baseline covariates in the censoring models were selected after systematic screening and comprised variables: age, sex, Charlson Comorbidity Index, Charlson comorbidity components (myocardial infarction, congestive heart failure, peripheral vascular disease, cerebrovascular disease, dementia, chronic pulmonary disease, rheumatic disease, peptic ulcer disease, mild liver disease, diabetes with and without complications, paraplegia, renal disease, malignant cancer, severe liver disease, metastatic solid tumor, AIDS), first-day summaries of vital signs (heart rate, systolic blood pressure, respiratory rate, SpO2), laboratory markers (lactate, creatinine, BUN, WBC, hemoglobin, platelets), mechanical ventilation, vasoactive agent use, and urine output. Severity scores (SOFA, APSIII, GCS, RASS, CAM-ICU) were not included in the censoring models because they are measured over the first 24 h (partly after exposure in early-treated patients) and were largely redundant with the direct physiological, laboratory, and intervention variables; ICU type was not included. Continuous covariates were standardized before model fitting to improve numerical stability. The standardized mean differences before and after IPCW weighting for each covariates is shown in Supplementary Table 3.

For binary outcomes, we estimated IPCW-weighted risks and risk differences and fit IPCW-weighted logistic regression models to obtain odds ratios. Confidence intervals and *p*-values for all primary and sensitivity estimates were obtained from patient-level nonparametric bootstrap (1,000 replicates for primary analyses; 500 for sensitivity analyses): at each replicate, patients were resampled with replacement, the entire clone-censor-weight chain (including censoring-model fitting and weight truncation) was re-estimated, and percentile intervals were computed. Resampling at the patient level accounts for within-patient correlation among ICU stays. For continuous outcomes (e.g., ICU LOS and restricted survival time), we compared IPCW-weighted means and mean differences; p -values were obtained using IPCW-weighted Mann–Whitney U tests or t -tests as appropriate.

For time-to-event outcomes, we generated IPCW-weighted Kaplan–Meier survival curves and fit IPCW-weighted Cox proportional hazards models to estimate hazard ratios (HR) with 95% confidence intervals. Survival distributions were compared using IPCW-weighted log-rank tests. For AECOPD, cumulative risk over time was visualized using cumulative incidence curves estimated using the Aalen-Johansen estimator, treating ICU discharge alive and death as competing events.

Several pre-specified sensitivity analyses were conducted. We varied the grace period (12, 24, and 36 h); considered alternative AECOPD definitions (systemic corticosteroids or antibiotics [primary definition]; systemic corticosteroids and antibiotics [joint definition, highest specificity]; systemic corticosteroids only; antibiotics only; systemic corticosteroids or COPD-specific antibiotics excluding broad-spectrum ceftriaxone and cefuroxime; and systemic corticosteroids and antibiotics initiated within ±24 h of each other); varied the exposure definition (inputevents records only [primary]; union with prescription records); restricted the cohort to the first ICU stay per patient; evaluated alternative censoring-model specifications (covariate sets; random forest and gradient boosting models); and examined alternative weight truncation thresholds (95th, 97th, and 99th percentiles).

Pre-specified subgroup analyses assessed effect modification by demographics, comorbidities, clinical characteristics (including mechanical ventilation, vasoactive agent use, and SpO2 strata), and laboratory strata (including creatinine, BUN, and lactate). Subgroup strata were restricted to pre-exposure baseline characteristics; severity scores measured over the first 24 h (SOFA, APSIII) were not used for stratification because they may be affected by sedation itself. Subgroup-specific associations were summarized using forest plots (IPCW-weighted 2 × 2 estimates with Woolf confidence intervals); these analyses are exploratory and should be interpreted cautiously due to multiple comparisons.

All analyses were performed using Python 3.12 (pandas, numpy, scipy, lifelines, statsmodels, scikit-learn, matplotlib). A two-sided *p* < 0.05 was considered statistically significant. For subgroup analyses, a more conservative threshold (*p* < 0.01) was used to partially account for multiple comparisons.

## Results

### Baseline characteristics

A total of 3,416 ICU stays were included; 233 (6.8%) received midazolam within 24 h of ICU admission and 3,183 (93.2%) did not (Figure 1). The treatment group differed from the control group in several baseline characteristics. The treatment group was slightly younger (median 70 [IQR 61–78] vs. 72 [IQR 64–79] years; *p* = 0.020) and had a higher proportion of male patients (62.7% vs. 54.6%; *p* = 0.020). Race distribution also differed, with fewer White patients (67.4% vs. 73.8%; *p* = 0.038) in the treatment group. Table 1 shows a detailed distribution of baseline characteristics.

**Figure 1:**
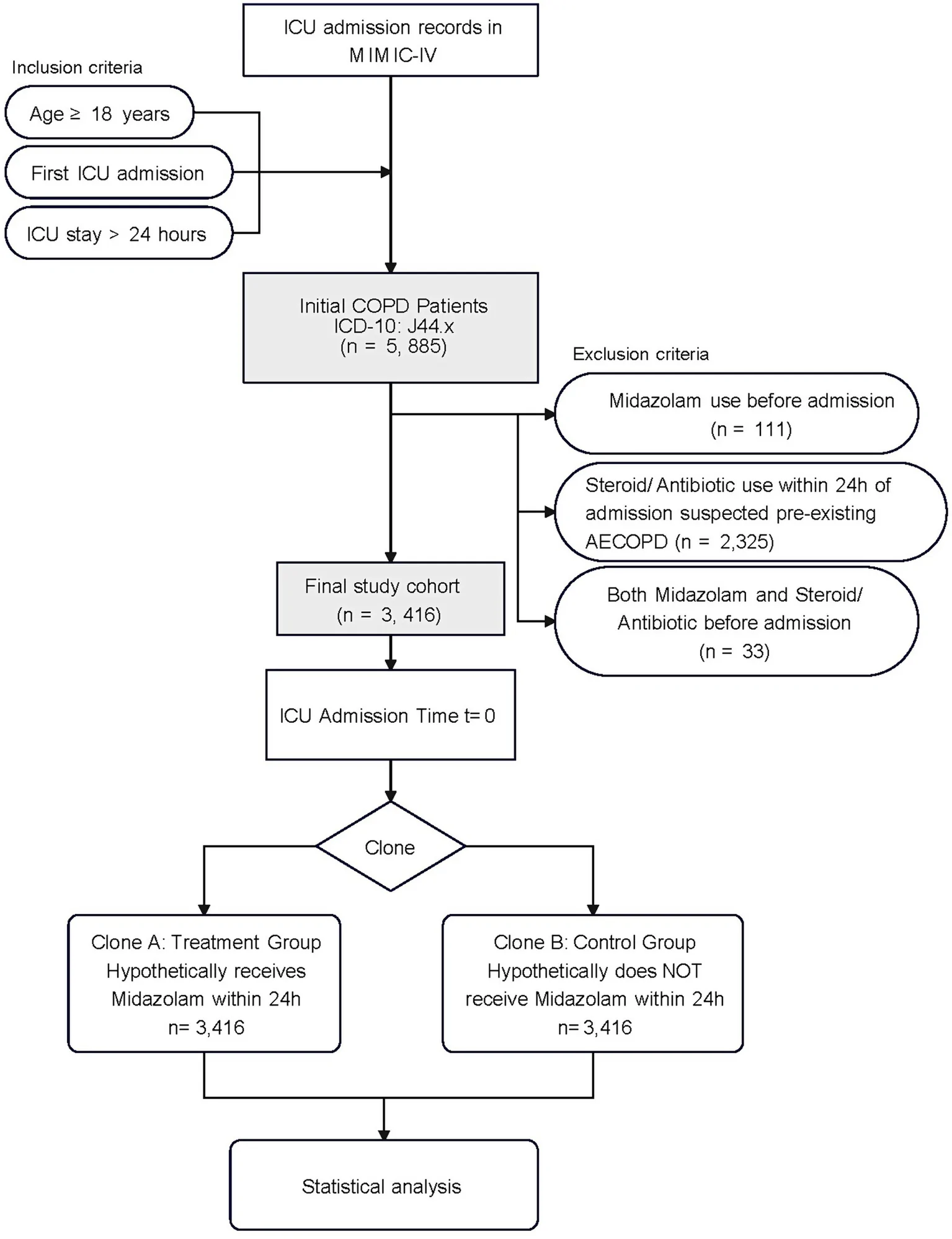
Inclusion and exclusion flowchart of the study. ICU, intensive care unit; MIMIC-IV, Medical Information Mart for Intensive Care IV; COPD, chronic obstructive pulmonary disease; AECOPD, acute exacerbation of COPD.

**Table 1:** Baseline characteristics of the cohort participants.

Variable	Total	Midazolam	Control	*p*-value	SMD (pre-weighting)	SMD (post-weighting)
*N*	3416	267	3149			
Demographics						
Age, years	72.0 (64.0–79.0)	70.0 (61.0–78.0)	72.0 (64.0–79.0)	0.020	−0.187	−0.041
Male, *n* (%)	1884 (55.2%)	146 (62.7%)	1738 (54.6%)	0.020	0.162	−0.051
Race						
White, *n* (%)	2507 (73.4%)	157 (67.4%)	2350 (73.8%)	0.038		
Black, *n* (%)	232 (6.8%)	16 (6.9%)	216 (6.8%)	1.000		
Others, *n* (%)	677 (19.8%)	60 (25.8%)	617 (19.4%)	0.023		
Severity scores						
SOFA score	1.0 (0.0–3.0)	2.0 (0.0–5.0)	1.0 (0.0–3.0)	<0.001	0.348	−0.039
APSIII score	42.0 (32.0–54.0)	47.0 (37.0–63.0)	42.0 (31.0–54.0)	<0.001	0.399	0.114
Charlson comorbidity index	7.0 (5.0–9.0)	7.0 (5.0–9.0)	7.0 (5.0–9.0)	0.177	−0.086	0.161
GCS (min)	15.0 (14.0–15.0)	15.0 (14.0–15.0)	15.0 (14.0–15.0)	<0.001	0.062	0.095
Urine output (first day), mL	1395.0 (879.8–2125.5)	1441.0 (850.0–2160.0)	1390.0 (880.0–2125.0)	0.864	0.068	0.008
RASS score (first 24 h)	0.0 (−3.0–0.0)	−2.0 (−4.0–0.0)	0.0 (−2.0–0.0)	<0.001	−0.545	−0.135
Delirium positive (CAM-ICU), *n* (%)	483 (19.4%)	58 (42.6%)	425 (18.1%)	<0.001	0.332	0.249
Vital signs (first day)						
Heart rate, bpm	82.3 (73.3–94.0)	83.8 (73.0–97.2)	82.2 (73.3–93.7)	0.093	0.122	0.255
Heart rate min, bpm	68.0 (60.0–79.0)	69.0 (59.0–80.0)	68.0 (60.0–79.0)	0.845	−0.006	0.189
Heart rate max, bpm	100.0 (88.0–115.0)	105.0 (91.0–124.0)	99.0 (88.0–115.0)	0.001	0.224	0.292
Systolic BP, mmHg	114.0 (105.6–125.4)	111.0 (104.3–120.6)	114.2 (105.6–125.9)	0.001	−0.246	−0.020
Diastolic BP, mmHg	60.7 (54.6–68.2)	60.0 (53.7–65.8)	60.7 (54.6–68.4)	0.031	−0.168	−0.046
Mean BP, mmHg	76.2 (70.6–83.8)	74.6 (69.6–81.2)	76.4 (70.7–84.0)	<0.001	−0.249	−0.093
Respiratory rate, /min	19.2 (17.0–21.7)	19.9 (17.5–22.8)	19.1 (16.9–21.6)	0.004	0.202	0.086
Temperature, °C	36.8 (36.6–37.0)	37.0 (36.7–37.3)	36.8 (36.6–37.0)	<0.001	0.592	0.456
SpO2, %	96.4 (94.9–97.8)	97.2 (95.7–98.6)	96.4 (94.9–97.7)	<0.001	0.289	0.178
SpO2 min, %	91.0 (88.0–93.0)	92.0 (88.0–95.0)	91.0 (88.0–93.0)	0.002	−0.014	−0.012
Laboratory (first day)						
WBC max, ×10⁹/L	12.6 (9.3–17.7)	15.8 (10.9–20.7)	12.4 (9.1–17.6)	<0.001	0.274	0.089
Hemoglobin min, g/dL	9.2 (7.8–11.0)	9.2 (7.7–10.9)	9.2 (7.8–11.0)	0.491	−0.054	−0.115
Platelet min, ×10⁹/L	174.0 (127.0–237.5)	157.0 (117.5–227.5)	174.0 (127.8–238.0)	0.038	−0.095	−0.059
Creatinine max, mg/dL	1.1 (0.8–1.8)	1.3 (0.9–2.1)	1.1 (0.8–1.8)	<0.001	0.098	0.010
BUN max, mg/dL	23.0 (15.0–39.0)	27.0 (19.0–45.0)	23.0 (15.0–39.0)	<0.001	0.137	−0.036
Sodium min, mEq/L	137.0 (134.0–140.0)	137.0 (134.0–140.0)	137.0 (134.0–140.0)	0.730	0.000	−0.049
Sodium max, mEq/L	139.0 (137.0–142.0)	140.0 (137.0–143.0)	139.0 (137.0–142.0)	0.027	0.149	−0.029
Potassium min, mEq/L	4.0 (3.7–4.4)	4.0 (3.6–4.6)	4.0 (3.7–4.4)	0.970	−0.035	−0.061
Potassium max, mEq/L	4.5 (4.1–5.1)	4.8 (4.3–5.5)	4.5 (4.1–5.0)	<0.001	0.396	0.356
Glucose max, mg/dL	140.0 (115.0–184.0)	155.0 (120.0–208.0)	140.0 (115.0–182.0)	0.002	0.121	−0.051
Lactate max, mmol/L	2.0 (1.3–3.1)	2.5 (1.4–3.9)	2.0 (1.3–3.0)	0.003	0.280	−0.031
Blood gas (arterial)						
pH min	7.3 (7.2–7.4)	7.3 (7.2–7.4)	7.3 (7.3-7.4)	0.010	−0.227	0.187
pH max	7.4 (7.4-7.5)	7.4 (7.3–7.5)	7.4 (7.4-7.5)	0.094	−0.146	−0.236
PaO2 min, mmHg	86.0 (70.0–113.0)	82.0 (70.0–113.0)	86.0 (71.0–113.0)	0.790	0.169	0.204
PaO2 max, mmHg	190.0 (102.0–360.0)	180.0 (114.5–358.5)	191.0 (99.0–359.8)	0.553	0.000	−0.315
PaCO2 min, mmHg	38.0 (34.0–44.0)	38.0 (33.5–43.0)	38.0 (34.0–44.0)	0.730	−0.093	0.166
PaCO2 max, mmHg	49.0 (42.0–57.0)	50.0 (42.0–58.0)	48.0 (42.0–57.0)	0.409	0.061	−0.131
Comorbidities						
Myocardial infarction, *n* (%)	843 (24.7%)	73 (31.3%)	770 (24.2%)	0.018	0.166	0.005
Congestive heart failure, *n* (%)	1690 (49.5%)	126 (54.1%)	1564 (49.1%)	0.165	0.099	0.011
Peripheral vascular disease, *n* (%)	754 (22.1%)	47 (20.2%)	707 (22.2%)	0.520	−0.049	−0.015
Cerebrovascular disease, *n* (%)	639 (18.7%)	44 (18.9%)	595 (18.7%)	1.000	0.005	0.128
Dementia, *n* (%)	218 (6.4%)	22 (9.4%)	196 (6.2%)	0.066	0.134	−0.028
Rheumatic disease, *n* (%)	130 (3.8%)	10 (4.3%)	120 (3.8%)	0.822	0.027	−0.014
Peptic ulcer disease, *n* (%)	128 (3.7%)	22 (9.4%)	106 (3.3%)	<0.001	0.323	0.049
Mild liver disease, *n* (%)	298 (8.7%)	23 (9.9%)	275 (8.6%)	0.601	0.044	0.174
Diabetes without complications, *n* (%)	856 (25.1%)	70 (30.0%)	786 (24.7%)	0.082	0.123	−0.130
Diabetes with complications, *n* (%)	766 (22.4%)	46 (19.7%)	720 (22.6%)	0.350	−0.069	0.041
Paraplegia, *n* (%)	210 (6.1%)	10 (4.3%)	200 (6.3%)	0.280	−0.083	−0.065
Renal disease, *n* (%)	1099 (32.2%)	67 (28.8%)	1032 (32.4%)	0.278	−0.078	0.212
Malignant cancer, *n* (%)	480 (14.1%)	31 (13.3%)	449 (14.1%)	0.809	−0.023	0.147
Severe liver disease, *n* (%)	148 (4.3%)	10 (4.3%)	138 (4.3%)	1.000	−0.002	0.047
Metastatic solid tumor, *n* (%)	174 (5.1%)	8 (3.4%)	166 (5.2%)	0.298	−0.081	−0.033
AIDS, *n* (%)	21 (0.6%)	1 (0.4%)	20 (0.6%)	1.000	−0.025	0.341
ICU information						
Neuro intermediate, *n* (%)	240 (7.0%)	5 (1.9%)	235 (7.5%)			
Cardiac vascular intensive care unit (CVICU), *n* (%)	689 (20.2%)	34 (14.6%)	655 (20.6%)			
Coronary care unit (CCU), *n* (%)	521 (15.3%)	40 (17.2%)	481 (15.1%)			
Surgical intensive care unit (SICU), *n* (%)	358 (10.5%)	20 (8.6%)	338 (10.6%)			
Medical intensive care unit (MICU), *n* (%)	579 (16.9%)	60 (25.8%)	519 (16.3%)			
Drugs and treatments (first day)						
Mechanical ventilation, *n* (%)	1237 (36.2%)	187 (80.3%)	1050 (33.0%)	<0.001	1.015	0.051
Antibiotic use, *n* (%)	1714 (50.2%)	163 (70.0%)	1551 (48.7%)	<0.001	0.427	0.088
Renal replacement therapy, *n* (%)	75 (2.2%)	8 (3.4%)	67 (2.1%)	0.269	0.091	−0.086
Any vasoactive agent, *n* (%)	1019 (29.8%)	156 (67.0%)	863 (27.1%)	<0.001	0.892	0.036
Norepinephrine, *n* (%)	825 (24.2%)	148 (55.4%)	677 (21.5%)	<0.001		
Dopamine, *n* (%)	44 (1.3%)	7 (3.0%)	37 (1.2%)	0.035	0.163	0.008
Dobutamine, *n* (%)	46 (1.3%)	8 (3.4%)	38 (1.2%)	0.010	0.194	0.240
Epinephrine, *n* (%)	143 (4.2%)	25 (10.7%)	118 (3.7%)	<0.001	0.352	0.014
Vasopressin, *n* (%)	159 (4.7%)	38 (16.3%)	121 (3.8%)	<0.001	0.600	0.036
Crystalloid volume, mL	646.3 (0.0–1880.9)	1632.3 (613.8–2830.7)	570.2 (0.0–1795.3)	<0.001	0.639	0.334
Colloid volume, mL	0.0 (0.0–0.0)	0.0 (0.0–0.0)	0.0 (0.0–0.0)	0.044	0.255	-0.016

Illness severity at baseline was higher among patients who received midazolam. SOFA scores were higher (median 2.0 [IQR 0.0–5.0] vs. 1.0 [IQR 0.0–3.0]; *p* < 0.001), and APSIII scores were substantially higher (47.0 [IQR 37.0–63.0] vs. 42.0 [IQR 31.0–54.0]; *p* < 0.001), with a higher APSIII probability of death (0.10 [IQR 0.06–0.19] vs. 0.08 [IQR 0.05–0.13]; *p* < 0.001). Charlson Comorbidity Index was similar between groups (median 7.0 [IQR 5.0–9.0] in both; *p* = 0.177). Although median GCS was 15.0 in both groups, the distribution differed (*p* < 0.001).

Several first-day physiological measurements differed. The treatment group had lower systolic blood pressure (111.0 vs. 114.2 mmHg; *p* = 0.001) and mean arterial pressure (74.6 vs. 76.4 mmHg; *p* = 0.001), slightly higher respiratory rate (19.9 vs. 19.1 breaths/min; *p* = 0.004), higher temperature (37.0 °C vs. 36.8 °C; *p* < 0.001), and higher SpO2 (97.2% vs. 96.4%; *p* < 0.001).

Laboratory markers were also more abnormal among exposed patients. The treatment group had higher maximum WBC (15.8 vs. 12.4 × 10^9^/L; *p* < 0.001), creatinine (1.3 vs. 1.1 mg/dL; *p* < 0.001), BUN (27.0 vs. 23.0 mg/dL; *p* < 0.001), potassium (4.8 vs. 4.5 mEq/L; *p* < 0.001), glucose (155.0 vs. 140.0 mg/dL; *p* = 0.002), and lactate (2.5 vs. 2.0 mmol/L; *p* = 0.003). Several comorbidities were more prevalent in the treatment group, including myocardial infarction (31.3% vs. 24.2%; *p* = 0.018) and peptic ulcer disease (9.4% vs. 3.3%; *p* < 0.001).

ICU management was more intensive among treated patients. Mechanical ventilation (80.3% vs. 33.0%; *p* < 0.001), antibiotic use (70.0% vs. 48.7%; *p* < 0.001), and vasoactive agent use (67.0% vs. 27.1%; *p* < 0.001) were all more common, and crystalloid volume was higher (median 1,632.3 vs. 570.2 mL; *p* < 0.001). Before weighting, baseline AECOPD occurrence (28.3% vs. 23.5%; *p* = 0.115) and 7-day mortality (9.4% vs. 6.8%; *p* = 0.154) did not differ significantly, whereas ICU length of stay was longer (median 4.5 vs. 2.7 days; *p* < 0.001), highlighting the need for weighting to account for baseline imbalance.

### Primary outcome

In the unweighted per-protocol comparison, the AECOPD incidence was 28.3% in the midazolam group versus 23.5% in controls (odds ratio 1.29, 95% CI 0.96–1.73). After IPCW weighting, the AECOPD incidence was 29.8% in the midazolam group versus 23.5% in controls, corresponding to a risk difference of +6.3% (95% CI −2.7% to +15.8%) and an odds ratio of 1.38 (95% CI 0.86–2.12; *p* = 0.158; Table 2). The absolute event counts were 66/233 unweighted clones (weighted 29.8%) in the midazolam group and 749/3,183 (weighted 23.5%) in the control group. The Aalen-Johansen cumulative incidence curves for AECOPD, treating ICU discharge alive and death as competing events, are shown in Figure 2A.

**Table 2:** Outcomes in patients with COPD in the ICU after clone-censor-weight (IPCW weighted).

Outcome	Grace period 12 h	Grace period 24 h	Grace period 36 h
Exposed, *n* (%)	190 (5.6%)	233 (6.8%)	255 (7.5%)
AECOPD unweighted, midazolam	30.0%	28.3%	29.4%
AECOPD unweighted, control	23.5%	23.5%	23.4%
AECOPD weighted, midazolam (95% CI)	33.4% (22.2–43.9%)	29.8% (21.0–39.1%)	28.2% (20.4–37.3%)
AECOPD weighted, control (95% CI)	23.5% (22.3–24.6%)	23.5% (22.3–24.6%)	23.3% (22.2–24.4%)
RD (95% CI)	+10.0% (−1.3 to 20.4)	+6.3% (−2.7 to 15.8)	+4.9% (−2.8 to 13.8)
*p*-value of RD	0.076	0.158	0.192
OR (95% CI)	1.64 (0.93–2.56)	1.38 (0.86–2.12)	1.29 (0.85–1.93)
*p*-value of OR	0.076	0.158	0.192
7 d mortality, midazolam (95% CI)	5.2% (2.0–10.2%)	4.1% (2.2–6.6%)	4.8% (2.8–7.4%)
7 d mortality, control (95% CI)	7.0% (6.3–7.7%)	6.9% (6.2–7.7%)	6.9% (6.2–7.7%)
28 d mortality, midazolam (95% CI)	8.6% (5.1–14.1%)	6.6% (4.6–9.6%)	10.6% (7.1–15.4%)
28 d mortality, control (95% CI)	13.5% (12.4–14.5%)	13.5% (12.4–14.5%)	13.4% (12.3–14.5%)
ICU LOS, midazolam (95% CI), d	5.4 (4.4–6.4)	5.5 (4.7–6.5)	5.6 (4.9–6.3)
ICU LOS, control (95% CI), d	4.8 (4.6–5.0)	4.8 (4.6–4.9)	4.7 (4.5–4.9)

**Figure 2:**
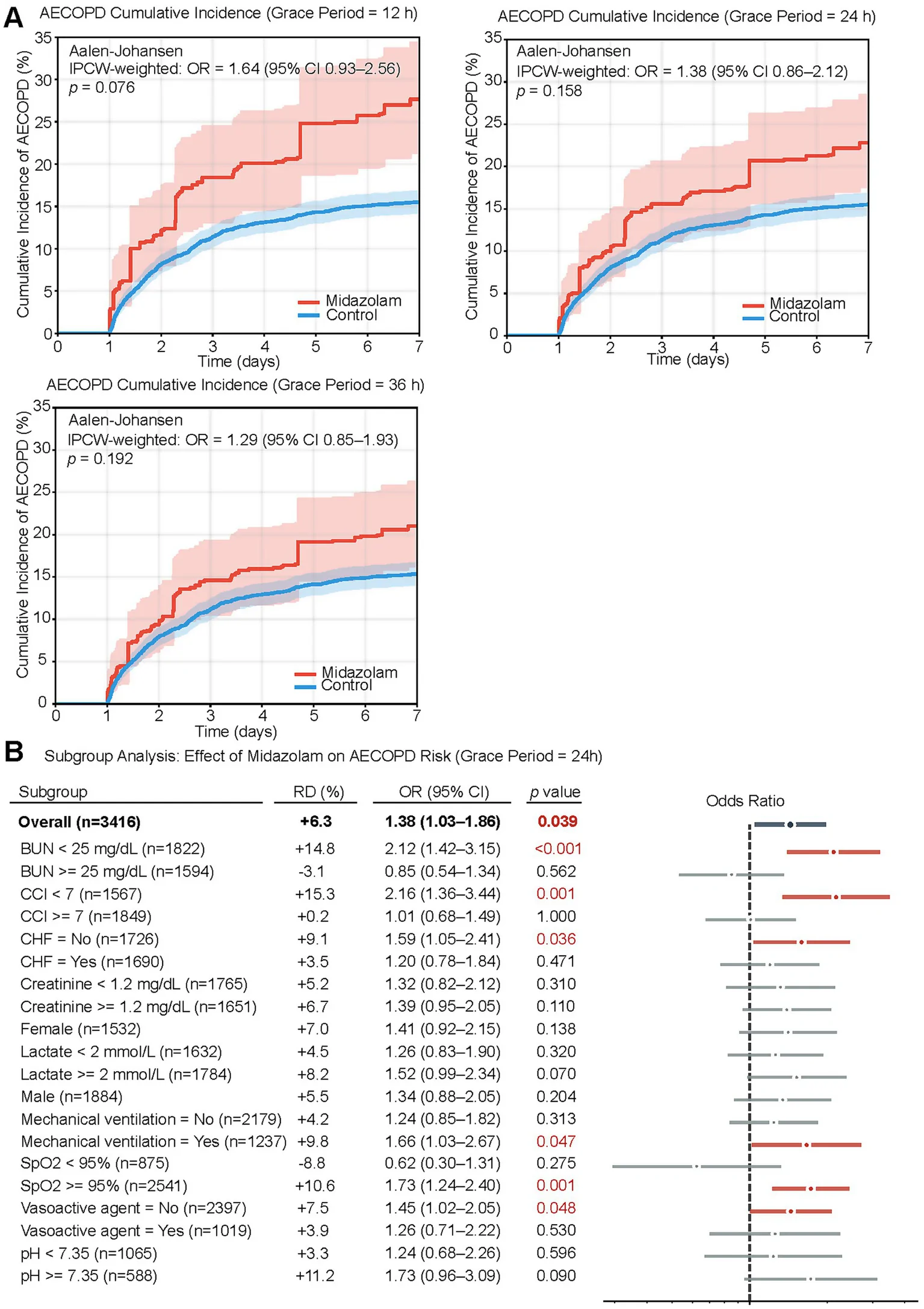
AECOPD cumulative incidence (Aalen-Johansen estimator, competing risks: ICU discharge alive, death) and forest plot for subgroup analysis after using the clone-censor-weight method. **(A)** AECOPD cumulative incidence at the 24 h grace period; OR, risk difference, and 95% CIs from patient-level bootstrap (1,000 replicates). **(B)** Forest plot of IPCW-weighted subgroup odds ratios (exploratory). AECOPD, acute exacerbation of COPD; CI, confidence interval; RD, risk difference; OR, odds ratio.

Subgroup analysis (Figure 2B) suggested larger point estimates among patients with lower baseline severity: BUN below 25 mg/dL (OR 2.12, 95% CI 1.42–3.15; risk difference +14.8%), SpO2 at least 95% (OR 1.73, 95% CI 1.24–2.40; risk difference +10.6%), and mechanically ventilated patients (OR 1.66, 95% CI 1.03–2.67; risk difference +9.8%). Larger estimates were also observed among patients without congestive heart failure (OR 1.59, 95% CI 1.05–2.41; risk difference +9.1%) and without vasoactive agent use (OR 1.45, 95% CI 1.02–2.05; risk difference +7.5%). Subgroup strata were restricted to pre-exposure baseline characteristics. These exploratory analyses should be interpreted cautiously due to multiple comparisons.

### Secondary outcomes

In weighted analyses, 7-day mortality was 4.1% (95% CI 2.2–6.6%) in the treatment group versus 6.9% (95% CI 6.2–7.7%) in controls (risk difference −2.8%; *p* = 0.034; Table 2). The weighted Kaplan–Meier survival curves for 7-day and 28-day survival are presented in Figure 3.

**Figure 3:**
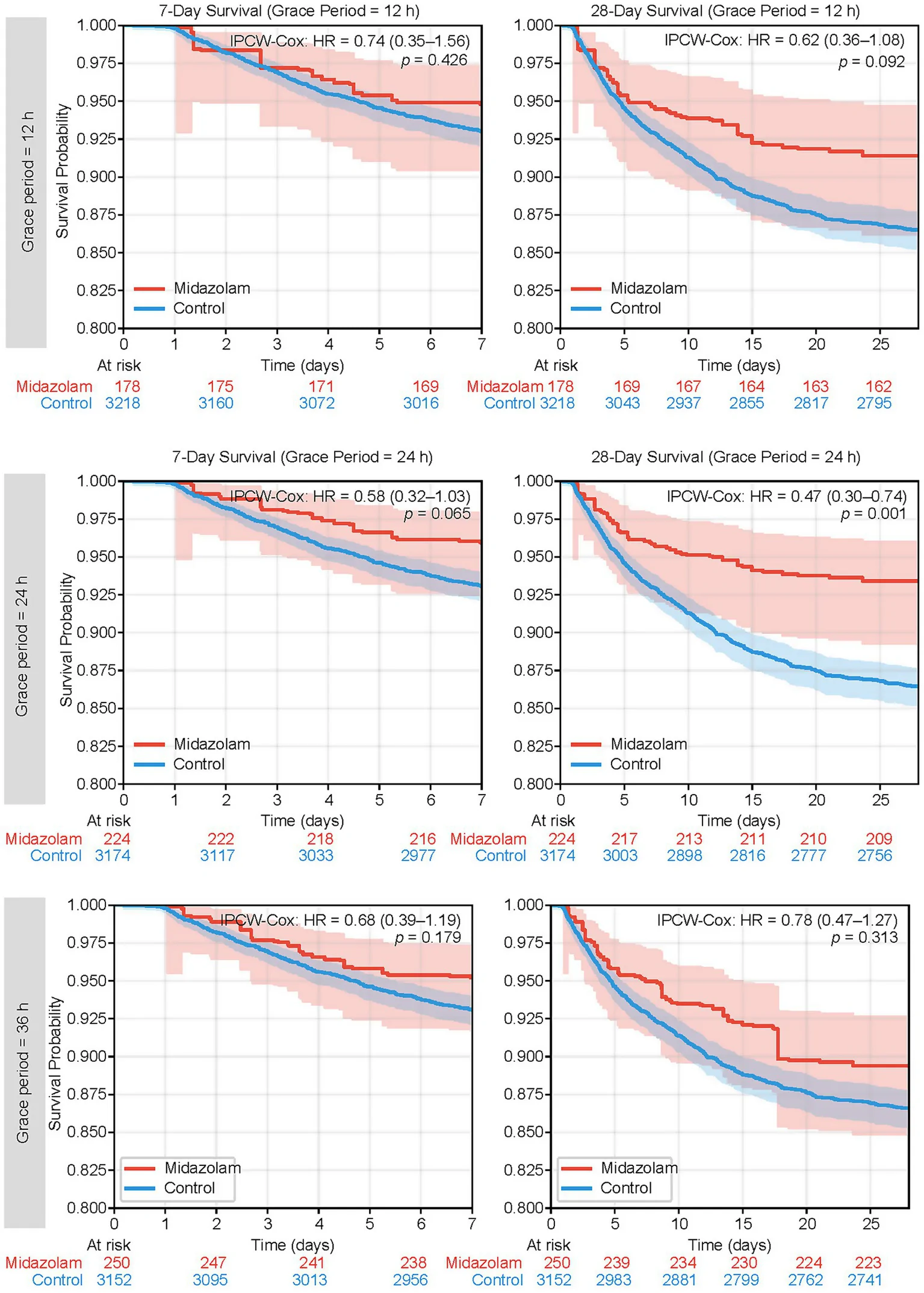
7-day and 28-day Kaplan–Meier survival curves after using the clone-censor-weight method. 7-day and 28-day survival at grace periods of 12 h, 24 h, and 36 h. Survival is estimated for time to death from ICU admission (recorded time of death) with administrative censoring at the horizon (day 7 or day 28); a 7-day cumulative incidence of death with ICU discharge alive as a competing event (Aalen-Johansen estimator) is also shown. CI, confidence interval; HR, hazard ratio. At-risk counts represent the IPCW-weighted effective sample size; values at t = 0 are the sum of IPCW weights (not raw patient counts).

The IPCW-weighted Cox proportional hazards model for time to death within 7 days of ICU admission suggested a lower hazard in the midazolam group that did not reach statistical significance (HR 0.58, 95% CI 0.32–1.03; *p* = 0.065), and the weighted log-rank test was non-significant (*p* = 0.099); at the 12 h and 36 h grace periods the corresponding HRs were 0.74 (95% CI 0.35–1.56; *p* = 0.426) and 0.68 (95% CI 0.39–1.19; *p* = 0.179). For 28-day mortality, the IPCW-weighted risk was 6.6% (95% CI 4.6–9.6%) in the treatment group versus 13.5% (95% CI 12.4–14.5%) in controls (risk difference −6.9%; *p* < 0.001), and the corresponding Cox HR was 0.47 (95% CI 0.30–0.74; *p* = 0.001). ICU length of stay was longer among treated patients: the IPCW-weighted mean ICU LOS was 5.5 days (95% CI 4.7–6.5) in the treatment group and 4.8 days (95% CI 4.6–4.9) in the control group, for a mean difference of +0.8 days (*p* = 0.078). They should be interpreted cautiously because longer ICU length of stay in the exposed group can mechanically increase observed “time under observation” without implying a survival benefit.

### Sensitivity analyses

To address potential limitations of the AECOPD definition, we performed sensitivity analyses using alternative outcome definitions (all at the 24 h grace period with identical weighting): corticosteroids AND antibiotics (joint definition; OR 1.80, 95% CI 0.48–3.20), corticosteroids only (OR 1.54, 95% CI 0.53–2.71), antibiotics only (OR 1.07, 95% CI 0.57–1.82), corticosteroids OR COPD-specific antibiotics excluding broad-spectrum agents (OR 1.70, 95% CI 0.96–2.65), and corticosteroids AND antibiotics initiated within ±24 h of each other (OR 1.02, 95% CI 0.08–2.09). None of the alternative definitions changed the conclusions (Supplementary Table 1).

Associations between midazolam and AECOPD were directionally consistent across alternative exposure definitions. With a 12 h grace period, 190 patients (5.6%) were classified as exposed. The IPCW-weighted AECOPD incidence was 33.4% in treated patients versus 23.5% in controls, corresponding to a risk difference of +10.0% (95% CI −1.3% to +20.4%; *p* = 0.076) and an odds ratio of 1.64 (95% CI 0.93–2.56). With a 36 h grace period, 255 patients (7.5%) were classified as exposed. The IPCW-weighted AECOPD incidence was 28.2% in treated patients versus 23.3% in controls, corresponding to a risk difference of +4.9% (95% CI −2.8% to +13.8%; *p* = 0.192) and an odds ratio of 1.29 (95% CI 0.85–1.93). The 12 h grace period showed the largest point estimate, suggesting that very early exposure may be particularly relevant to short-term exacerbation risk, although none of the grace-period analyses reached statistical significance.

## Discussion

In this study, we emulated a target trial using the MIMIC-IV database to evaluate the association between early ICU midazolam exposure and short-term AECOPD risk in critically ill COPD patients using a CCW framework with a corrected IPCW implementation. In the primary analysis (24 h grace period), early midazolam exposure was associated with a numerically higher AECOPD risk, but the association did not reach statistical significance, and the wide confidence intervals reflect the limited number of exposed patients (*n* = 233, 6.8%). Directionally consistent point estimates were observed across the 12 h (OR 1.64) and 36 h (OR 1.29) grace periods, with none reaching statistical significance. These findings do not establish that midazolam causes AECOPD; they indicate that an association cannot be excluded and should be examined in studies designed to estimate causal effects.

These findings are broadly consistent with ICU sedation guidance and with prior ICU sedation evidence that has raised concerns about benzodiazepine-based sedation. The SCCM PADIS guidelines recommend minimizing benzodiazepine exposure when possible and often favor non-benzodiazepine sedatives for many mechanically ventilated adults (Devlin et al., 2018). Although these recommendations were not designed specifically for COPD, they are supported by evidence linking benzodiazepine exposure to delirium and by syntheses suggesting worse ICU process outcomes compared with non-benzodiazepine strategies (Fraser et al., 2013; Pandharipande et al., 2006; Pandharipande et al., 2007). Randomized ICU sedation trials have compared dexmedetomidine with midazolam or propofol and reported improved patient interaction and, in some comparisons, shorter ventilation duration (Jakob et al., 2012; Riker et al., 2009). Protocols emphasizing daily sedation interruption and paired spontaneous awakening/breathing trials have also been shown to shorten ventilation duration and improve ICU outcomes (Girard et al., 2008; Kress et al., 2000). In large observational analyses of mechanically ventilated patients from MIMIC-IV, midazolam was associated with prolonged mechanical ventilation, whereas dexmedetomidine-containing regimens showed more favorable adjusted mortality (Shi et al., 2024).

Several mechanisms could link early midazolam exposure to COPD worsening. Benzodiazepines can depress central respiratory drive and reduce upper airway tone; in susceptible COPD phenotypes, this may promote hypoventilation and hypercapnia and increase the work of breathing after sedation interruption (Agusti and Vogelmeier, 2023). Sedation can also impair cough and secretion clearance and may contribute to atelectasis or infectious complications that prompt initiation of systemic corticosteroids and/or antibiotics. In addition, sedation choice may indirectly shape ventilator management. COPD ventilation requires careful avoidance of dynamic hyperinflation and patient-ventilator asynchrony; deeper or prolonged sedation may delay weaning readiness or obscure early signs of deterioration.

The relationship between confounding by indication and our estimates deserves emphasis. Patients who received midazolam were substantially sicker at baseline (mechanical ventilation 80.3% vs. 33.0%; vasoactive agents 67.0% vs. 27.1%; higher APSIII), and severity was strongly associated with both exposure and outcome. The unweighted per-protocol comparison (OR 1.29, 95% CI 0.96–1.73) therefore reflects a comparison of sicker treated patients with healthier controls. Our corrected IPCW implementation reweights treated patients toward the characteristics of the full study population (per-arm censoring models; down-weighting typical recipients and up-weighting atypical recipients), and post-weighting standardized mean differences improved substantially for the strongest confounders (mechanical ventilation 1.02 to 0.05; vasoactive agents 0.89 to 0.04). Nonetheless, the point estimate moved only modestly, the confidence intervals remained wide, and residual imbalance persisted for several covariates, so residual confounding by indication remains a central limitation.

Unmeasured confounding is also likely. Pre-admission COPD severity (spirometry, GOLD stage), smoking history, home oxygen use, chronic hypercapnia, and the clinical indication for sedation are not recorded in MIMIC-IV. Because these factors are associated with both the propensity to receive midazolam and the risk of respiratory deterioration, they could bias the estimate in either direction. Quantitatively, an unmeasured confounder with relative risk 1.90 for both exposure and outcome would be sufficient to fully explain the unweighted association (*E*-value = 1.90 for OR 1.29); measured confounders of comparable or greater strength (e.g., mechanical ventilation) exist, underscoring the plausibility of residual confounding. Conversely, if midazolam is preferentially used in patients with less severe baseline exacerbation burden, the true association could be underestimated. The *E*-value for the adjusted OR of 1.38 is 2.11 (for the CI lower bound 0.86, the *E*-value is 1.00 because the interval includes the null), indicating the fragility of the point estimate.

The primary endpoint (AECOPD) is a medication-based proxy. The combination of systemic corticosteroids and antibiotics has a positive predictive value above 75–85% for true exacerbations in validation studies of electronic health records, whereas either drug class alone performs less well (Rothnie et al., 2016). In our sensitivity analyses, the joint definition (highest specificity) yielded the largest point estimate (OR 1.80), suggesting that outcome non-specificity does not inflate the observed association. Conversely, corticosteroids and antibiotics are frequently used for non-exacerbation indications in the ICU (sepsis, pneumonia), which could attenuate the association through outcome misclassification; we mitigated this by excluding patients already receiving these medications within the first 24 h and by the antibiotic-specific definitions. Direct chart-based outcome adjudication was not possible.

IPCW-weighted mortality risks were lower in the treated group in the 7-day analysis and the 28-day analysis, while ICU length of stay was longer among treated patients. This pattern—lower mortality but longer ICU stay, together with a numerically higher medication-based exacerbation rate—should be interpreted cautiously: it is consistent with residual selection (patients who received midazolam represent a group that tolerated early sedation), with longer exposure to ICU observation, and with earlier reports of longer ventilation duration under midazolam-based sedation. It also reinforces that the medication-based AECOPD endpoint captures treatment escalation rather than clinical deterioration alone. Hazard-based comparisons may be distorted by short follow-up, non-proportionality, and competing events such as ICU discharge. The observed heterogeneity across subgroups provides additional clinical hypotheses. We observed larger point estimates in patients with lower baseline comorbidity and severity burden (lower Charlson Comorbidity Index, lower BUN, higher SpO2, absence of congestive heart failure, absence of vasoactive agents), which may reflect a higher relative contribution of midazolam’s respiratory depressant effects in patients whose clinical trajectory is less dominated by their underlying acute illness, and higher risk among mechanically ventilated patients, consistent with the role of sedation in prolonging ventilator dependence and impeding secretion clearance. These patterns should be interpreted as hypothesis-generating and require validation in independent cohorts.

Strengths of this study include the explicit target trial specification, the CCW/IPCW design to reduce immortal time bias, and a fully reproducible analytical pipeline in which the censoring models, weighting, and bootstrap inference are re-estimated within a single framework. The final censoring model incorporates 33 baseline covariates, and the sensitivity analyses span outcome definitions, exposure definitions, cohort restrictions, weight truncation, machine-learning censoring models, and alternative covariate specifications. Limitations include: (1) potential misclassification of AECOPD when defined by medication initiation as a proxy, particularly in the ICU setting where corticosteroids and antibiotics are frequently used for non-exacerbation indications; (2) residual confounding by unmeasured variables, including pre-admission COPD severity, smoking history, home oxygen use, sedation indication, and ventilator management details; (3) the heterogeneous control group, which precludes head-to-head comparisons between specific sedative agents; (4) the lack of a validated dose–response analysis, as MIMIC-IV infusion data did not reliably distinguish bolus from continuous administration; (5) limited statistical precision because only 6.8% of the cohort received midazolam within 24 h; and (6) limited generalizability beyond a single health system.

## Summary

In this target trial emulation among critically ill COPD patients, early ICU midazolam exposure was associated with a higher short-term AECOPD risk, but the difference was not statistically significant. Because the present study did not demonstrate a statistically significant causal effect, its results alone cannot justify a change in clinical practice, and the caution expressed here is informed primarily by the existing ICU sedation evidence. These observational findings, together with existing ICU sedation evidence and guideline recommendations to minimize benzodiazepine use, support continued caution in the use of midazolam in this population and motivate confirmatory studies, including randomized evidence, comparing sedative strategies in COPD patients.

## Data Availability

The original contributions presented in the study are included in the article/Supplementary material, further inquiries can be directed to the corresponding author.
