# Supplementary material for: Early midazolam exposure and short-term risk of acute exacerbation of COPD in critically ill patients: a target trial emulation using the MIMIC-IV database
Source: Front Artif Intell. 2026 Oct 6;9:1836581. doi: 10.3389/frai.2026.1836581 (PMC13680442; doi:10.3389/frai.2026.1836581)
Supplement: Supplementary file 1 [file Supplementary_file_1.zip › Supplementary file 1/Supplementary Material/Supplementary File 1.DOCX]

**Supplemental Table 1.** Sensitivity analyses using alternative outcome definitions: corticosteroids AND antibiotics (joint), corticosteroids only, and antibiotics only (IPCW-weighted, Grace Period = 24h).

| **Analysis** | **OR** | **OR 95% CI** | **RD %** | **RD 95% CI** | **P value** |
| --- | --- | --- | --- | --- | --- |
| S1: steroid AND antibiotic (joint) | 1.80 | 0.48-3.20 | 2.55 | -1.7 to 6.6 | 0.41 |
| S2: steroid only | 1.54 | 0.53-2.71 | 3.47 | -3.4 to 10.1 | 0.27 |
| S3: antibiotic only | 1.07 | 0.57-1.82 | 0.76 | -5.2 to 8.1 | 0.80 |
| S4: steroid OR COPD-specific antibiotic | 1.70 | 0.96-2.65 | 8.57 | -0.6 to 17.6 | 0.07 |
| S5: steroid AND antibiotic within 24h | 1.02 | 0.08-2.09 | 0.03 | -1.9 to 2.1 | 0.63 |
| S6: first ICU stay per patient | 1.30 | 0.73-2.10 | 5.25 | -5.4 to 16.0 | 0.34 |

**Supplemental Table 2.** TTE protocol of the study.

| Protocol Element | Description |
| --- | --- |
| Eligibility criteria | Adults ≥18 years with COPD (ICD-10 J44.x), first ICU stay, ICU LOS >24h. Excluded: prior midazolam, early steroids/antibiotics (<24h), missing key variables. |
| Treatment strategies | Strategy 1: Initiate midazolam within grace period (12h/24h/36h) after ICU admission. Strategy 2: Do not initiate midazolam within grace period. |
| Assignment procedures | Non-randomized observational comparison. CCW method: each patient cloned to both strategies; strategy-inconsistent clones censored; IPCW applied. |
| Outcomes | Primary: AECOPD (steroids or antibiotics, 24h–7d). Secondary: 7d/28d mortality, ICU LOS, RMST/RMTL. |
| Follow-up | From ICU admission (time zero) to AECOPD, death, ICU discharge, or 7 days (primary). 28-day window for mortality. |
| Causal contrast | Per-protocol effect: estimated effect of adhering to each strategy, adjusted for informative censoring via IPCW. |
| Analysis plan | IPCW-weighted logistic regression for binary outcomes; Cox PH models for survival; Aalen-Johansen CIF for competing risks; Fine-Gray models. Expanded model: 34 covariates, multiple imputation, stabilized weights. |

**Supplemental Table 3. Standardized mean differences before and after IPCW weighting (GP = 24 h, 33 covariates)**

| Covariate | SMD pre-weighting | SMD post-weighting |
| --- | --- | --- |
| Age | -0.187 | -0.041 |
| Male sex | 0.162 | -0.051 |
| Charlson index | -0.086 | 0.161 |
| Heart rate (mean) | 0.122 | 0.255 |
| SBP (mean) | -0.246 | -0.020 |
| Resp rate (mean) | 0.203 | 0.086 |
| SpO2 (mean) | 0.288 | 0.178 |
| Mechanical ventilation | 1.015 | 0.051 |
| Vasoactive agents | 0.893 | 0.036 |
| Lactate (max) | 0.242 | -0.031 |
| Creatinine (max) | 0.100 | 0.010 |
| BUN (max) | 0.132 | -0.036 |
| WBC (max) | 0.276 | 0.089 |
| Hemoglobin (min) | -0.054 | -0.115 |
| Platelets (min) | -0.095 | -0.059 |
| Urine output | 0.073 | 0.008 |
| Myocardial infarction | 0.166 | 0.005 |
| Congestive heart failure | 0.099 | 0.011 |
| Peripheral vascular disease | -0.049 | -0.015 |
| Cerebrovascular disease | 0.005 | 0.128 |
| Dementia | 0.134 | -0.028 |
| Chronic pulmonary disease | 0.000 | 0.000 |
| Rheumatic disease | 0.027 | -0.014 |
| Peptic ulcer disease | 0.323 | 0.049 |
| Mild liver disease | 0.044 | 0.174 |
| Diabetes (uncomplicated) | 0.124 | -0.130 |
| Diabetes (complicated) | -0.069 | 0.041 |
| Paraplegia | -0.083 | -0.065 |
| Renal disease | -0.079 | 0.212 |
| Malignant cancer | -0.023 | 0.147 |
| Severe liver disease | -0.002 | 0.047 |
| Metastatic solid tumor | -0.081 | -0.033 |
| AIDS | -0.025 | 0.341 |

Figure S1. Positivity assessment — propensity score distribution in the midazolam and control groups, demonstrating adequate overlap.

Figure S2. Love plot showing standardized mean differences for all 34 covariates before and after IPCW weighting. The dashed red lines indicate the conventional balance threshold of ±0.1.

Figure S3. IPCW weight distribution for the treatment and control groups after stabilization and truncation at the 99th percentile.
