# Supplementary material for: Early midazolam exposure and short-term risk of acute exacerbation of COPD in critically ill patients: a target trial emulation using the MIMIC-IV database
Source: Front Artif Intell. 2026 Oct 6;9:1836581. doi: 10.3389/frai.2026.1836581 (PMC13680442; doi:10.3389/frai.2026.1836581)
Supplement: Supplementary file 1 [file Supplementary_file_1.zip › Supplementary file 1/Supplementary Material/Table 1.DOCX]

| Item | Description | Reported on |
| --- | --- | --- |
| **Abstract** |  |  |
| 1a | Study identified as an observational emulation of a target trial; objectives and brief summary of the specified target trial | Abstract |
| 1b | Data sources used for emulation | Abstract (MIMIC-IV v3.1) |
| 1c | Key assumptions, statistical methods, findings, conclusions | Abstract |
| **Introduction** |  |  |
| 2 | Scientific background and knowledge gap | Introduction |
| 3 | Causal question summarized | Introduction (final paragraph) |
| 4 | Rationale for emulating a target trial with available data | Introduction |
| **Methods** |  |  |
| 5 | Data sources: original purpose, type, location, setting, time period | Methods, “Study design and data source” |
| 6 | Target trial specification: eligibility, treatment strategies, assignment, follow-up, outcomes, causal contrasts, identifying assumptions | Methods (TTE specification) |
| 7 | Emulation: how each target trial component was operationalized with the data | Methods (CCW procedure, exposure/outcome definitions) |
| **Results** |  |  |
| 8 | Population flow (numbers eligible, included, excluded) | Results + Figure 1 |
| 9 | Baseline characteristics of included patients | Results + Table 1 |
| 10 | Follow-up duration and outcome event counts | Results + Table 2 |
| 11 | Estimates of each causal contrast with measures of precision | Results, Primary/Secondary outcomes + Table 2 |
| 12 | Results of analyses assessing identifying assumptions (positivity, balance) | Results + Supplemental Tables 3 |
| 13 | Sensitivity analyses addressing design/analysis choices | Results, Sensitivity analyses + Supplemental Table 2 |
| 14 | Subgroup/effect-modification analyses (if any) | Results, Subgroup analysis + Figure 2B |
| **Discussion** |  |  |
| 15 | Interpretation consistent with results; consideration of assumptions and limitations | Discussion |
| 16 | Generalizability | Discussion, Limitations |
| **Other information** |  |  |
| 17 | Registration and protocol availability | N/A (observational database study; analysis plan prespecified in Methods) |
| 18 | Sources of funding and role of funders | Funding statement |
| 19 | Competing interests | Conflict of interest statement |
| 20 | Data availability | Data availability statement (MIMIC-IV PhysioNet) |
| 21 | Code availability | Data availability statement (code available upon reasonable request) |
